# A Novel Blocking Enzyme-Linked Immunosorbent Assay Based on a Biotinylated Nanobody for the Rapid and Sensitive Clinical Detection of Classical Swine Fever Virus Antibodies

**DOI:** 10.1128/spectrum.02996-22

**Published:** 2023-01-23

**Authors:** Zhi Cao, Dehua Yin, Lihong Zhang, Shuai Ma, Ke Zhang, Ruimei Yang, Hu Shan, Zhihua Qin

**Affiliations:** a College of Veterinary Medicine, Qingdao Agricultural University, Qingdao, China; b Qingdao Animal Disease Prevention and Control Center, Qingdao, China; Suranaree University of Technology

**Keywords:** classical swine fever virus, E2 recombinant protein, nanobody, blocking ELISA

## Abstract

Monoclonal and polyclonal antibodies are mostly used for the development of traditional enzyme-linked immunosorbent assays (ELISAs), but the use of certain conventional antibodies may be limited by their low yield, the difficulty of their isolation, and their high cost. Heavy-chain antibodies derived from camelids with naturally missing light chains can overcome these deficiencies and are an excellent alternative to conventional antibodies. In this study, a nanobody (Nb)-AviTag fusion protein was constructed, and the feasibility of its use as a high-sensitivity probe in a blocking ELISA (bELISA) for classical swine fever virus (CSFV) was investigated. The CSFV E2 recombinant protein expressed by the CHO expression system exhibited good reactogenicity and immunogenicity and induced the production of high CSFV antibody levels in rabbits. Three different clones of Nbs were successfully isolated using a phage display system in alpaca, and an Nb1-AviTag fusion protein was successfully expressed using an Escherichia coli expression system. The purified Nb1-AviTag fusion protein was then biotinylated *in vitro* to obtain Nb1-biotin. A novel bELISA was developed for the detection of CSFV antibodies in clinical serum using Nb1-biotin as a probe. The cutoff value of bELISA was 32.18%, the sensitivity of bELISA was higher than that of the bELISA kit with IDEXX antibody, and the coincidence rate was 94.7%. A rapid, low-cost, highly sensitive and highly specific CSFV E2 antibody-based bELISA method was successfully established and can be used for the serological evaluation of CSFV E2 subunit vaccines and the ELISA-based diagnosis of CSFV infection.

**IMPORTANCE** Currently, the epidemic situation of classical swine fever (CSF) is sporadic, and cases of atypical swine fever are on the rise in China. Therefore, it is necessary to accurately eliminate suspected cases by using highly sensitive and specific diagnostic techniques. In our study, a rapid, low-cost, highly sensitivity, highly reliable and reproducible, and highly specific classical swine fever virus (CSFV) E2 antibody-based blocking ELISA method was successfully established by using the phage display system and the Nb1-AviTag fusion expression platform. It provides a new technique for serological evaluation of CSFV vaccines and ELISA-based diagnosis of CSFV infection.

## INTRODUCTION

Classical swine fever (CSF) is a highly contagious and fatal infectious disease of pigs caused by classical swine fever virus (CSFV) (1). Although some countries have eliminated CSF through the use of vaccines and other means, more than 40 countries and regions in the world, including China, still suffer from CSF epidemics (2–4). Some countries that previously declared that they had eradicated CSF are suffering from a resurgence of the disease (5). In China, CSF is currently a local sporadic, recessive infection with a long incubation period (6). Therefore, highly sensitive detection methods can detect CSF earlier and prevent large-scale outbreaks and the import of viruses from abroad.

As recorded in the WOAH Terrestrial Manual, the diagnostic methods for CSF are the following: virus isolation, fluorescent antibody test (FAT), reverse transcription-PCR (RT-PCR), virus neutralization test (VNT), and antigen and antibody enzyme-linked immunosorbent assay (ELISA) (7–11). Among these methods, ELISA is widely used for clinical diagnosis, vaccine antibody evaluation, and differential diagnosis, among other uses (12–14). Conventional monoclonal and polyclonal antibodies are currently the standard, but these antibodies used in ELISA kits have high development costs, long production cycles, and short shelf lives and often do not truly meet the specificity and sensitivity requirements for clinical testing (15–17). Therefore, the development of an antibody that can be produced rapidly in large quantities and at a low price and that exhibits high specificity and a long shelf life is of great significance for ELISA detection.

Nanobodies (Nbs) are also known as the variable domain of the heavy chain of heavy-chain antibodies (VHHs) (18). Due to their lack of light chains, Nbs confer many special properties, including high affinity, thermal stability, and high yield, in microbial production systems (19–22). Nbs have been widely used in double-antibody sandwich ELISA, competitive ELISA, and blocking ELISA (bELISA) (23–25). The application of Nbs can improve the specificity and sensitivity of ELISA detection, and the occurrence of a disease can be detected earlier (26, 27). CSFV-specific Nbs have not yet been reported. In this study, the E2 protein of CSFV was expressed as an immune antigen through a mammalian expression system, phage display technology was used to obtain specific Nbs against the E2 protein of CSFV, and a novel bELISA based on a biotinylated Nb was developed for the rapid and sensitive clinical detection of CSFV antibodies.

## RESULTS

### Expression of CSFV E2 protein in CHO cells.

**FIG 1:**
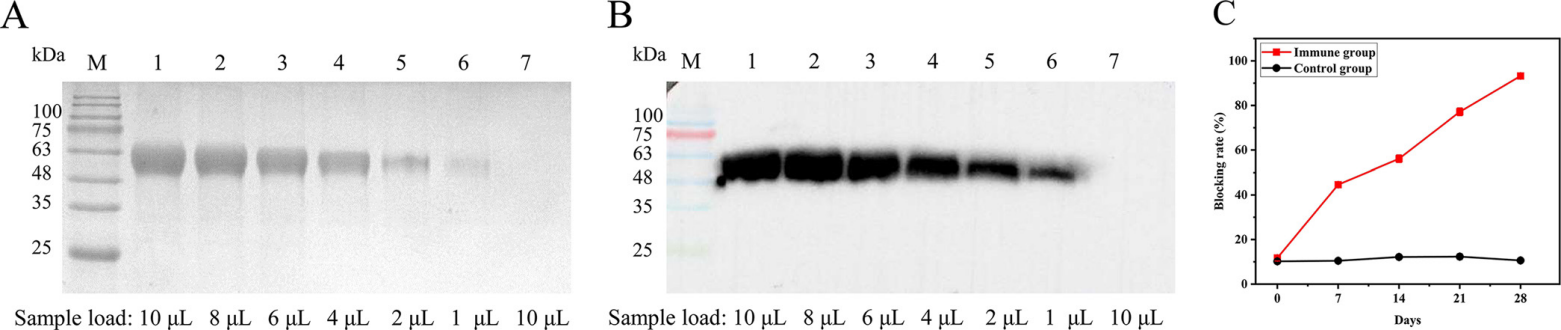
Expression and identification of the CSFV E2 protein. (A) Results of 12% SDS-PAGE. Lane M, prestained protein markers; lanes 1 to 6, transfected cells; lane 7, untransfected normal cells. (B) Results of WB. Lane M, prestained protein markers; lanes 1 to 7 are the same as described for panel A. (C) Validation of the immunogenicity of the CSFV E2 protein.

As validated by 12% sodium dodecyl sulfate-polyacrylamide gel electrophoresis (SDS-PAGE) and Western blotting (WB), the CSFV-E2 protein was obtained in good purity and exhibited good reactivity and a size of approximately 51 kDa, as expected (Fig. 1A and B). The serum was tested using an ELISA kit with CSFV antibody (IDEXX). The blocking rate increased with increases in the number of immunizations (Fig. 1C), indicating that the expressed recombinant protein exhibits high immunogenicity and could be used for the immunization of alpaca and the screening of specific Nbs.

### VHH library construction.

The purified E2 recombinant protein was used for the immunization of adult male alpaca, and serum from immunized alpaca was collected 1 week after the fifth immunization. The antibody titer was measured by indirect ELISA (iELISA), and the results indicated that the specific antibody titer reached 1:128,000 (see Fig. S1A in the supplemental material). One week after the last immunization, 20 mL of noncoagulated blood was collected, and a total of 2.8 × 10^7^ lymphocytes were isolated. Total RNA was extracted, and cDNA was obtained by reverse transcription. After two rounds of amplification by nested PCR, a target band of approximately 400 bp was obtained (Fig. S1B and C). The target band was recovered, cloned into the phage display vector pCANTAB 5E, and electrotransferred into TG1 competent cells to obtain a VHH phage display library with a library volume of 8 × 10^7^ (Fig. S1D). The transformant was identified by PCR of the bacterial solution. The results showed that 90% of the clones contained the target gene (the expected size of the amplified product was approximately 750 bp) (Fig. S1E).

### Specific Nb panning.

**FIG 2:**
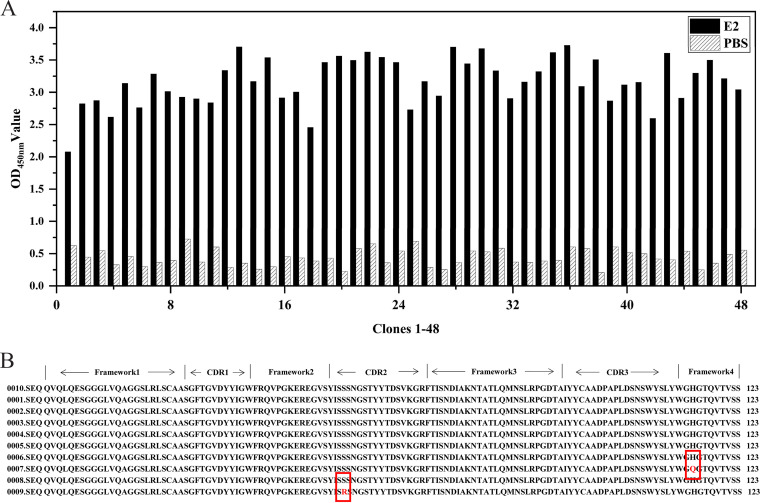
Screening Nbs against CSFV E2 protein. (A) Analysis of the binding ability of recombinant Nbs against E2 by iELISA. (B) Functional region division and amino acid sequence analysis of 10 Nbs.

The E2 recombinant protein was used as the coating antigen for the screening of specific Nbs. The results are shown in Table 1. The phage recovery rate increased gradually during three rounds of screening, and the ratio of the phage titers from the positive wells (*P* output) to those from the negative wells (*N* output) after the third round of screening was 1,059, which indicated that the recombinant phage was significantly enriched. The iELISA results indicated that all 48 selected clones were positive (Fig. 2A).

**TABLE 1:** Enrichment of phage particles carrying E2-specific Nbs^*a*^

Round of panning	Input phage (PFU/well)	*P* output (PFU/well)	*N* output (PFU/well)	Recovery (*P*/input)	*P* output/*N* output
1st	5 × 10^11^	3.4 × 10^7^	1.7 × 10^7^	6.8 × 10^−5^	2
2nd	5 × 10^11^	2.9 × 10^8^	2.6 × 10^6^	5.8 × 10^−4^	145
3rd	5 × 10^11^	2.86 × 10^9^	2.7 × 10^6^	5.7 × 10^−3^	1,059

a *P* output, the phage titers from the positive wells (CSFV E2); *N* output, the phage titers from the negative wells (PBS).

The 10 positive clones with the highest absorbance identified by ELISA were selected for sequencing analysis and were classified based on the amino acid sequence of the antibody hypervariable region. The 10 selected positive clones contained a total of three different Nbs (GenBank accession no. OP583736, OP583737, and OP583738), and the Nb with the highest number of amino acid sequence repeats was selected for expression (Fig. 2B).

### Specific Nb expression.

**FIG 3:**
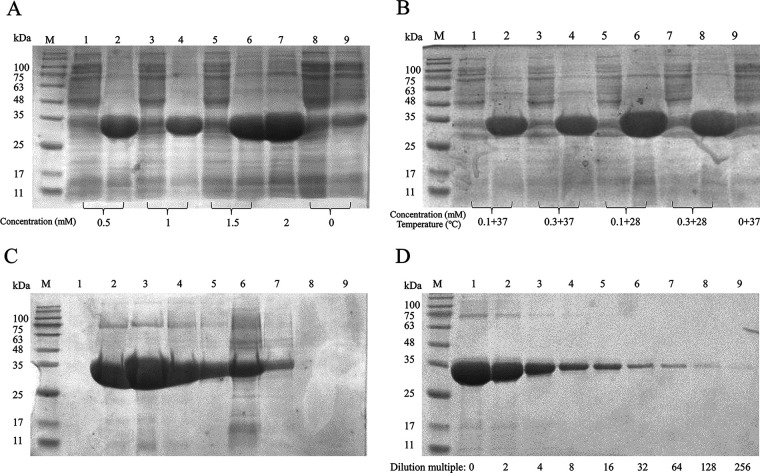
Expression of the pET32a-Nb1-AviTag fusion protein. (A) Expression results of Nb1-AviTag at different IPTG final concentrations. Lane M, prestained protein markers; lanes 1, 3, 5, and 8, supernatant; lanes 2, 4, 6, 7, and 9, precipitate. (B) Expression results of Nb1-AviTag at different final IPTG concentrations and induction temperatures. Lane M, prestained protein markers; lanes 1, 3, 5, and 7, supernatant; lanes 2, 4, 6, 8, and 9, precipitate. (C) Analysis of protein purification. Lane M, prestained protein markers; lanes 1 to 5, eluate after purification; lane 6, flowthrough; lanes 7 to 9, wash. (D) Analysis of double dilutions of lanes 1 to 9 after dialysis. Lane M, prestained protein markers.

Expression was induced using the checkerboard method with final IPTG (isopropyl-β-d-thiogalactopyranoside) concentrations of 0.5, 1, 1.5, and 2 mM and an induction time of 4 h. After ultrasonic crushing and centrifugation, the supernatant was collected, and the precipitate was resuspended in phosphate-buffered saline (PBS). Verification by 12% SDS-PAGE revealed obvious target bands near 34 kDa in the precipitate (Fig. 3A); thus, the target protein was inclusion body protein (Fig. 3B).

The expression of a large amount of Nb1-AviTag recombinant protein was induced. After sonication, a Ni resin affinity chromatography column was used for the purification of His-tagged Nb1-AviTag recombinant protein in the precipitate. The Ni resin had a strong binding capacity for Nb1-AviTag protein, resulting in good purity after purification (Fig. 3C). After purification, the protein was denatured in urea and then dialyzed using dialysates containing different concentrations of urea to obtain the soluble protein dissolved in PBS (Fig. 3D). The expression of Nb1-AviTag reached 137.9 mg/L.

### Establishment of a bELISA for CSFV antibody detection using the Nb1-AviTag fusion protein.

**FIG 4:**
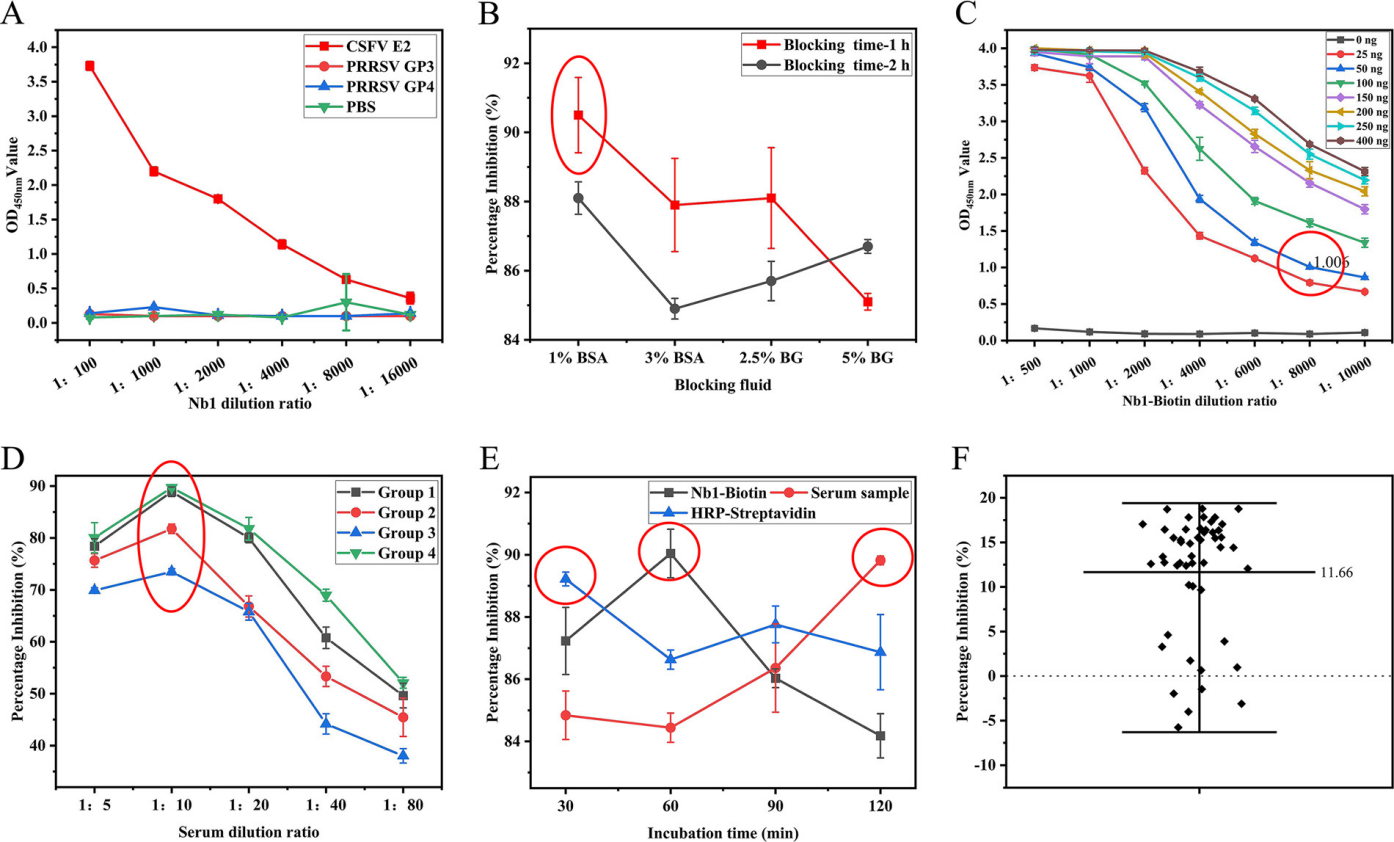
Optimized reaction conditions for the developed bELISA. (A) Nb1-biotin titer. (B) Screening of optimal blocking solutions. (C) Screening of optimal dilutions of CSFV E2 protein and Nb1-biotin. (D) Screening of optimal serum dilution. (E) Screening of optimal serum incubation, Nb1-biotin incubation, and HRP-streptavidin incubation times. (F) Distribution of the PI values obtained from the detection of CSFV antibody-negative porcine sera with the developed bELISA method.

The purified and renatured Nb1-AviTag fusion protein was biotinylated, and the iELISA results indicated that the maximum detected dilution of Nb1-biotin was 1:16,000 (Fig. 4A), which indicated that the Nb1-biotin and E2 proteins exhibit good binding abilities and can be used for the development of high-sensitivity ELISA kits for CSFV detection.

The optimization of the optimal blocking solution and blocking time showed that the optimal blocking effect was obtained after blocking with 1% bovine serum albumin (BSA) for 1 h, which meets the requirements for ELISAs (Fig. 4B).

By use of the checkerboard titration method, the optimal antigen coating volume and dilution ratio for the Nb were explored. The results showed that an antigen coating volume of 50 ng and a dilution ratio for Nb1-biotin of 1:8,000 yielded an optical density at 450 nm (OD_450_) of 1.0, which reveals that these are the optimal antigen coating amount and the optimal antibody dilution ratio (Fig. 4C). The results from the optimization of the serum dilution indicated that the control serum exhibited the highest blocking rate at a dilution of 1:10 (Fig. 4D).

Using the above-mentioned optimized conditions, the optimal incubation times for the serum to be tested, Nb1-biotin, and enzyme-labeled secondary antibody were explored. As demonstrated by ELISAs, the optimal incubation times for the serum to be tested, Nb1-biotin, and horseradish peroxidase (HRP)-streptavidin were 120 min, 60 min, and 30 min, respectively (Fig. 4E).

Cutoff values are critical with regard to the sensitivity of blocking ELISAs. The average percentage inhibition (PI) of the 50 negative serum samples was 11.66%, and the standard deviation (SD) was 6.84%. Therefore, a sample PI of >32.18% (11.66% + 3SD) indicated that the sample could be considered positive, and 25.34% < sample PI < 32.18% indicated that the sample should be considered suspicious and that reexamination is needed. If the reexamination result was still suspicious after reexamination, the sample was considered negative (Fig. 4F).

### Specificity, sensitivity, and repeatability of the developed bELISA.

**FIG 5:**
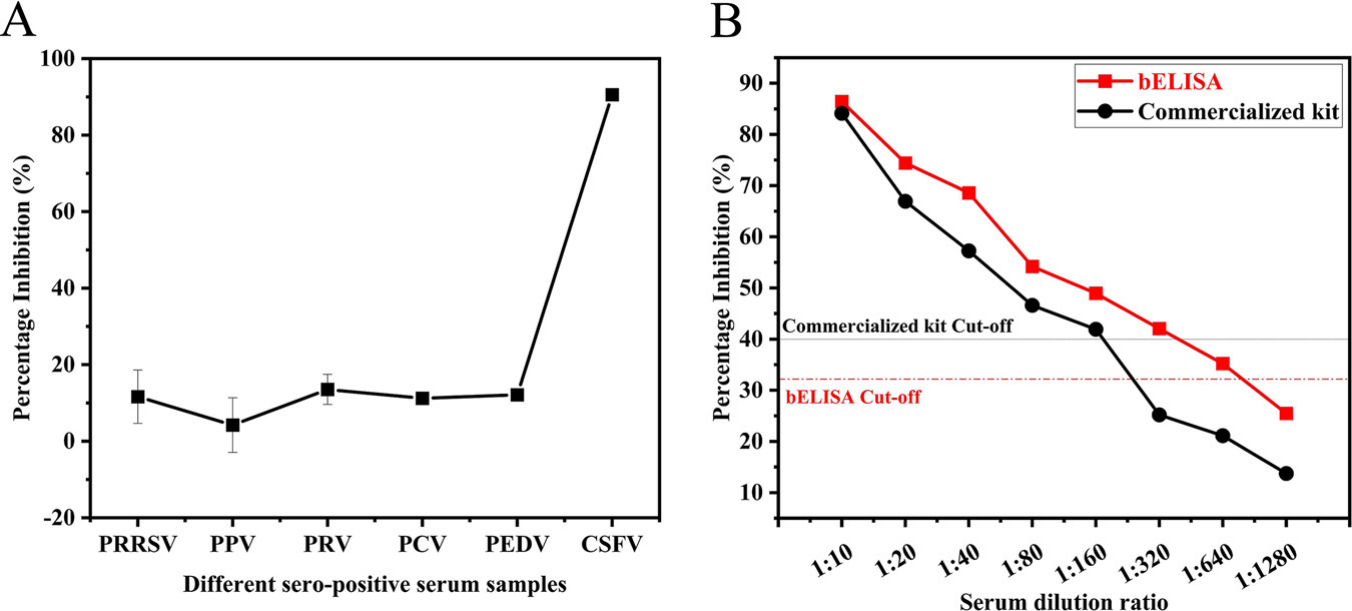
Sensitivity and specificity of the developed bELISA using CSFV Nb1-biotin as a probe. (A) Evaluation of the specificity of the developed bELISA method. (B) Evaluation of the sensitivity of the developed bELISA method.

To verify the specificity of the established bELISA method, serum samples positive for porcine reproductive and respiratory syndrome virus (PRRSV), pseudorabies virus (PRV), porcine parvovirus (PPV), porcine circovirus type (PCV), porcine epidemic diarrhea virus (PEDV), and CSFV were assessed, and the results are shown in Fig. 5A. The blocking rates for serum samples positive for PRRSV, PRV, PPV, PCV, PEDV, and CSFV were 11.6%, 4.2%, 13.5%, 11.2%, 12.1%, and 90.6%, respectively. The results indicate that the bELISA method exhibited no cross-reactivity and had strong specificity.

To verify the sensitivity of the detection method established in this study, clinically positive serum was diluted 2-fold from 1:10 to 1:1,280 and detected using the established method and a commercial reagent kit (IDEXX, USA; catalog no. 99-43220). The results obtained with the commercial reagent kit were no longer positive at dilutions of at least 1:320, whereas the bELISA method developed in this study yielded positive results with sample dilutions up to 1:640 (Fig. 5B).

Ten known positive clinical serum samples were used to evaluate the intraplate and interplate reproducibility of the bELISA method. As shown in Table 2, the intraplate variation (coefficient of variation [CV]) ranged from 0.72% to 4.63%, and the interplate variation (CV) ranged from 1.71% to 5.55%, which indicated that the detection method exhibited excellent repeatability.

**TABLE 2:** Repeatability evaluation

Repeatability	CV (%) range for 10 positive serum samples	Median CV (%)
Intraplate^*a*^	0.72–4.63	2.675
Interplate^*b*^	1.71–5.55	3.63

a The values were obtained from three replicates of 10 serum samples on the same microplate plate.

b The values were calculated from the values for 10 serum samples in three microplate plates.

### Validation of the developed bELISA.

For clinical samples, the results obtained with bELISA exhibited 94.7% agreement with those obtained with the commercial CSFV antibody-based bELISA (IDEXX, USA; catalog no. 99-43220) (Table 3). Moreover, based on the above-described detection results, the kappa value of the bELISA with the commercial CSFV antibody-based bELISA was 0.88, indicating good reproducibility.

**TABLE 3:** Comparison of blocking ELISA and commercial ELISA kit using clinical samples^*a*^

Sample type	Blocking ELISA result	No. of samples	Commercial ELISA kit result (no. of samples)		Agreement (%)	Kappa value
			Positive	Negative		
Clinical porcine serum	Positive	204	188	16	94.7	0.88
	Negative	96	0	96		

a The kappa value represents the consistency of the established bELISA and commercial ELISA (IDEXX, USA; 99-43220) results: 0.0 to 0.20, slight consistency; 0.21 to 0.40, fair consistency; 0.41 to 0.60, moderate consistency; 0.61 to 0.80, substantial consistency; and 0.81 to 1, almost perfect consistency.

## DISCUSSION

Despite decades of efforts, the elimination of CSF in epidemic-affected areas and areas of reemergence remains difficult (28). Rapid and accurate clinical diagnosis is particularly important as the first line of defense for the detection and control of primary CSF outbreaks (29). In serological diagnosis, antibody-based ELISA has the advantages of being rapid and simple (30). CSFV E2 protein can induce the host to produce neutralizing antibodies, and studies on the E2 protein subunit vaccine have shown that this protein can distinguish between vaccinated and infected animals (DIVA vaccine) (2). The combination of antibody-based ELISA and the DIVA vaccine is one method for eliminating CSF. Several companies have developed ELISA kits for CSFV antibody detection based on the CSFV E2 protein, and examples of these companies include Biocheck (catalog no. SK106 CSFV E2), Cusabio Technology LLC (catalog no. CSB-E12765p), IDEXX Laboratories (catalog no. 99-43220), ID VET (catalog no. CSFE2C-5P), and Thermo Fisher Scientific (catalog no. 7610046) (1). This finding indicates the feasibility of developing CSFV antibody detection methods based on the CSFV E2 protein, and these kits are based on the use of conventional antibodies as ELISA probes.

In this study, we screened a strain of Nb1 with high specificity for CSFV E2 using phage display technology and expressed the Nb1-AviTag fusion protein using a prokaryotic expression system, and the expression level reached 137.9 mg/mL, which is very considerable. Nb1-AviTag can be connected with biotin to yield Nb1-biotin. Many studies have shown that the application of biotin as an antibody probe in ELISA can improve the sensitivity and stability of detection (31, 32). We provide the first description of the successful development and validation of a bELISA method based on CSFV E2 protein Nbs for antibody detection in swine serum. In comparison with ELISA methods based on conventional antibodies, the use of Nbs improved the sensitivity and specificity and reduced the cost of the corresponding ELISAs.

Compared with more sensitive quantitative PCR (qPCR) assays, ELISAs can be used not only for the diagnosis of disease but also for the monitoring of antibody levels in individuals or populations and for the timely assessment of the immune effects of vaccination and population resistance (33). The bELISA developed in this study did not cross-react with PRRSV-, PRV-, PPV-, PCV-, or PEDV-seropositive samples, showing good specificity. The same positive sample was diluted twice and tested using a commercial kit (IDEXX, USA; catalog no. 99-43220) and bELISA. With a 1:1,280 dilution, the results of the established bELISA method were positive, and the results obtained with the commercial kit were negative, which indicated that the established bELISA method had higher sensitivity. The results showed that the coincidence rate of the positive samples was 100%, and the overall coincidence rate was 94.7%. Based on CSFV E2, Ji et al. and Li et al. developed an indirect ELISA method for CSFV antibody detection and found that its coincidence rate with the IDEXX 99-43220 kit was 92.2% and 80.3%, respectively, which was lower than the value of 94.7% found in this study (34, 35). The calculated kappa value of 0.88 was greater than 0.81, which shows that the established bELISA method is highly consistent with commercial kits and can be used for the detection of CSFV antibodies.

Several studies have shown that Nbs can inhibit virus replication (36–38). E2 is the most immunogenic protein of CSFV and can produce clinical protective antibodies against CSFV challenge (1). In this study, we inoculated the virus into PK-15 cells and added Nb1-AviTag to the maintenance solution to determine whether Nb1-AviTag inhibited virus replication, and the viral titers at 24 and 48 h were measured by *Taq*Man qPCR. Unfortunately, the experimental results showed that Nb1-AviTag exerted a certain inhibitory effect on viral replication, and this finding might have been obtained because Nb1-AviTag lacks a transmembrane peptide, which results in a poor inhibitory effect (37). Nbs can also be used in double-antibody sandwich ELISA, colloidal gold immunochromatography, and other detection methods, which have high sensitivity and specificity (39). In terms of treatment, most studies of Nbs have focused on the mechanism of the antiviral action of Nbs, which remain far from being sufficiently effective in clinical application (40). With increasing research, Nbs will accelerate the contribution to human public health and provide significant contributions to human public health.

### Conclusions.

In this study, a mammalian expression system was used to express the CSFV E2 recombinant protein for the immunization of alpaca, and a total of three different specific Nbs were screened and exhibited high affinity for the antigen protein. One Nb was selected for successful establishment of a bELISA method for CSFV, and the therapeutic efficacy of the assay was validated. A recombinant Nb fusion (Nb1-AviTag) protein was expressed. The bELISA detection method for CSFV established herein based on the expression of the Nb1-AviTag fusion protein was found to exhibit high specificity and sensitivity, have a low cost, be easily commercialized, and detect CSFV regardless of the species.

## MATERIALS AND METHODS

### Serum samples.

Standard CSFV-negative and CSFV-positive sera were provided in the ELISA kit (IDEXX, USA; catalog no. 99-43220), and serum samples of PRRSV, PCV, PRV, PPV, and PEDV were preserved in our laboratory. A total of 300 clinical serum samples from immunized pigs were collected from large farms in Shandong.

### Antigen preparation.

The pcDNA 3.4 TOPO-E2 plasmid was constructed based on the whole-genome sequence of the CSFV strain published by NCBI (pcDNA 3.4 TOPO and CSFV E2 gene ligation), and the CSFV E2 protein was expressed by the CHO mammalian expression system. The size and reactivity of the CSFV E2 protein were verified by 12% SDS-PAGE and Western blot (WB) assay. Anti-His antibody (Cell Signaling Technology, USA; catalog no.12698S) was used as the primary antibody, and goat anti-rabbit IgG H+L (HRP) (Abcam, UK; ab205718) served as the secondary antibody. After the protein was mixed with water-in-oil (W/O) emulsifier ISA 201 adjuvant (SEPPIC, France; 70142-34-6), the rabbit was immunized, the serum was collected, and the immunogenicity of the expressed protein in serum was detected with the CSFV antibody enzyme-linked immunosorbent test kit (IDEXX, USA; 99-43220).

### VHH library construction.

A healthy adult male alpaca was immunized with 5 mL of CSFV E2 recombinant protein (1 mg/mL) emulsified with W/O emulsifier ISA 201 adjuvant (SEPPIC, France; 70142-34-6). A total of five immunizations were administered at 2-week intervals. Serum was collected 1 week after each immunization for testing. The titer was measured by iELISA; peroxidase-conjugated AffiniPure goat anti-alpaca IgG H+L (Stratech, UK; 128-035-003-JIR) was used as the secondary antibody, 20 mL of noncoagulated blood was collected at the highest titer, and lymphocytes were then isolated. Total RNA (Vazyme, China; RC112-01) was extracted and reverse transcribed into cDNA (Vazyme, China; R323-01). Two pairs of primers, primers F1 and R1 and primers F2 and R2, were designed based on the IgG and heavy-chain antibodies (alpacas) in NCBI (41). By using cDNA as a template, PCR amplification was performed using primers F1 and R1 (see Table S1 in the supplemental material). The 700-bp target band was recovered from the gel, and the concentration of the product was determined. The product recovered from the gel was used as the template for PCR amplification using primers F2 and R2. The 400-bp band was recovered from the gel, and the concentration of cDNA was determined. Digestion was performed using the restriction endonucleases PstI and NotI, and the product and pCANTAB-5E vector were recovered from the gel. The target gene and vector were ligated using T4 ligase. After purification of the ligated product, the target recombinant plasmid was obtained. The target recombinant plasmid was electrotransferred into E. coli TG1 electrocompetent cells, and the cells were spread on Luria–Bertani (LB) agar plates containing 4% (vol/vol) glucose and 100 μg/mL ampicillin (LB/AMP-GLU) and incubated at 37°C for 12 h. Colonies were then scraped and stored in LB containing 20% glycerol at −80°C. The size and diversity of the library were evaluated by calculating the number of colonies and the insertion rate by PCR amplification (using primers F3 and R2) after gradient dilution, and the positive clones were sequenced.

### Specific Nb panning.

**FIG 6:**
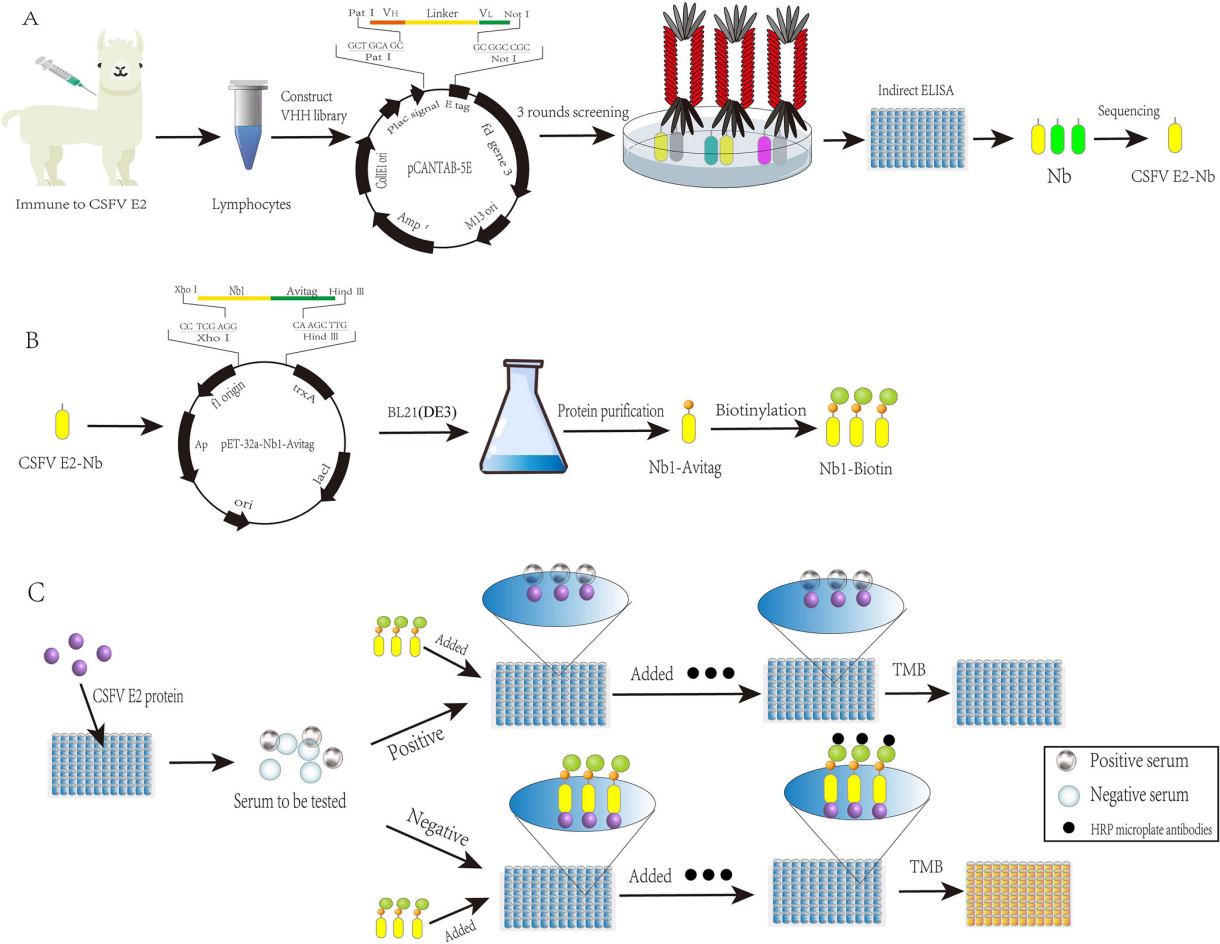
Graphical abstract. (A) Diagram of the acquisition of the VHH library. (B) Expression of the CSFV-Nb1-AviTag fusion protein. (C) Design of the developed bELISA.

For the selection of specific Nbs against the CSFV E2 protein, we performed phage rescue and titration as previously described (31, 41). The main method used for CSFV E2 protein-specific Nb panning was ELISA-specific adsorption for solid-phase panning (Fig. 6A). Briefly, the experimental wells in an ELISA plate were coated with purified CSFV E2 protein, and wells coated with PBS were used as controls. After four washes, the samples were blocked with 1% BSA. After four additional washes, 100 μL (5 × 10^12^ PFU/mL) of phage was added to each well, and the plate was incubated for 2 h. One hundred microliters of 0.1 M triethylamine was added, and the samples were incubated for 10 min. The liquid was collected by pipetting, and the same volume of 1 M Tris-HCl (pH 7.4) was added for neutralization. After the separate collection of the recombinant bacteriophages, the phages were diluted from 10^−1^ to 10^−9^ times using LB liquid medium, mixed with TG1 bacteria in the logarithmic phase of growth at a ratio of 1:1 (100 μL each), and incubated at 37°C for 30 min. Subsequently, 100 μL of the phages was incubated in LB/AMP-GLU plates at 37°C for 12 h, and the phage titers collected from the positive (*P* output) and negative (*N* output) wells were calculated separately based on the number of individual colonies. The enrichment of specific recombinant bacteriophages was determined by the ratio of the titer of recombinant phages eluted from positive pores to that from negative pores. Four hundred microliters of positive well-eluted recombinant bacteriophage was mixed with 4 mL of TG1 bacterial solution in the logarithmic phase of growth, incubated at 37°C for 30 min, transferred to 80 mL of LB medium containing 4% (vol/vol) glucose and 100 μg/mL ampicillin, and shaken vigorously at 200 rpm at 37°C until the bacterial culture at OD_600_ reached approximately 0.6. M13KO7 at an MOI of 20 was added to amplify the recombinant bacteriophage, and the amplified product was applied to the next solid phase. Once the enrichment was greater than 10^3^, the panning was considered completed. Expression of the 48 monoclonal colonies whose titer was measured for the third time was induced. After three cycles of freezing and thawing, the supernatant was collected. The positive clones were identified by iELISA. Anti-E tag antibody (Abcam, UK; ab3397) was used as the primary antibody, and goat anti-rabbit IgG H+L (HRP) (Abcam, UK; ab205718) served as the secondary antibody. The bacterial solution of the positive clones was sent for sequencing (i.e., the OD_450_ was more than 3 times greater than that of the PBS control), and the sequencing results were analyzed.

### Expression of specific Nbs.

Based on the sequencing results, the sequence to be expressed was selected, and the AviTag peptide was introduced into the 3′ end for biotin labeling. The pET-32a-Nb1-AviTag plasmid was synthesized by Sangon Biotech (Shanghai) Co., Ltd. The obtained recombinant plasmid was transformed into E. coli BL21(DE3) competent cells, and positive clones were selected and induced with different final concentrations of IPTG (0.5, 1, 1.5, and 2 mM). Each group was incubated at 37°C and 220 rpm for 4 h. The medium in which the bacteria were induced was collected by centrifugation, sonicated to disrupt the precipitate, centrifuged, and stored separately. Subsequently, 12% SDS-PAGE was performed to confirm the induced expression of the Nb1-AviTag fusion protein and the solubility of the recombinant Nb1-AviTag protein. The expressed protein was purified using Ni resin, and the eluate was collected (Fig. 6B). The purification of the protein was assessed by 12% SDS-PAGE.

The purified Nb1-AviTag protein is an inclusion protein dissolved in 8 M urea with no associated biological activity. Nb1-AviTag was reinstated by using dialysate bags and dialysis composed of urea and PBS, and the Nb1-AviTag protein after reduction was collected from PBS containing 6 M, 4 M, 2 M, and 1 M urea to PBS containing 0 M urea. Each urea concentration was dialyzed at 4°C for 12 h. A bicinchoninic acid (BCA) protein assay kit (Thermo Fisher, USA; A53226) was used to measure the protein concentration after renaturation, and the protein content was calculated.

### Application of purified Nbs in establishment of an ELISA detection method based on CSFV Nb blocking.

First, we biotinylated the renatured protein to facilitate development of the bELISA kit. Biotin ligase and the required buffer and Nb1-AviTag components were added to the microtube, and after addition of the sample, the tube was incubated at 30°C for 60 min to obtain biotinylated Nb1-AviTag (Nb1-biotin). The biotinylation effect was validated by iELISA using microplates coated with 400 ng of CSFV E2 protein, PRRSV GP3 and GP4 proteins diluted 2-fold from 1:100 to 1:16,000 with Nb1-biotin, horseradish peroxidase-labeled streptavidin (HRP-streptavidin) as the secondary antibody, and 3,3′,5,5′-tetramethylbenzidine (TMB) as the color rendering solution. The OD_450_ was read using a microplate reader to assess the specificity of Nb1-biotin (Fig. 6C).

Moreover, 1% and 3% BSA and 2.5% and 5% (wt/vol) nonfat dried milk (blotting grade [BG]) powder were tested to determine the optimal blocking solution and blocking time. ELISA detection was performed after blocking for 1 and 2 h. The optimal protein coating amount and the degree of Nb dilution were determined using the checkerboard method. During coating, CSFV E2 protein was diluted to 0, 0.25, 0.5, 1, 1.5, 2, 2.5, and 4 μg/mL, and Nb1-biotin was diluted 1:500, 1:1,000, 1:2,000, 1:4,000, 1:8,000, and 1:10,000 for ELISA detection. An OD_450_ of 1 revealed the optimal combination (31).

To determine the optimal serum dilution ratio, four CSF-seropositive serum samples and four CSF-seronegative serum samples diluted at a 2-fold ratio (1:5 to 1:80) were used for bELISA detection, and the blocking rate (PI value) of the four groups of serum samples was calculated using the following formula: percent inhibition (PI) = (1 − [*P*/*N*]) × 100%, where *P* and *N* are the ODs of the seropositive and seronegative serum samples, respectively. Furthermore, the optimal incubation time for the serum sample was determined. The serum sample was diluted based on the optimal dilution ratio and incubated for 30, 60, 90, and 120 min for ELISA detection. The PI ratios were calculated. The optimal incubation times for Nb1-biotin and the enzyme-labeled secondary antibody (HRP-streptavidin) were determined using the same method.

Fifty known negative serum samples were selected to determine the cutoff value for the bELISA method. Three replicates of each well were included based on the optimal reaction conditions determined previously. The PI ratios were calculated. The cutoff value equals the mean PI plus 3 SDs (31).

### Evaluation of the bELISA method.

The established ELISA method was used for the detection of PRRSV, PCV, PRV, PPV, and PEDV in the positive-control serum samples. Three replicates of each serum sample were included, and the results were statistically analyzed to evaluate the specificity of the established method.

The known positive serum samples from pigs were serially diluted from 1:10 to 1:1,280 by double dilution, and the established bELISA method and a commercial reagent kit (IDEXX, USA; 99-43220) were used for detection. Three technical replicates of each serum sample were included, and the results were statistically analyzed to evaluate the sensitivity of the established method.

Ten known positive serum samples were selected to evaluate the reproducibility of the established bELISA method based on the differences within and between plates. Ten positive serum samples were subjected to ELISA detection, and the CV of the PI value within a plate was calculated. Ten positive serum samples were tested using different batches of coated ELISA plates at three different times. The CV of the PI values between plates was calculated.

### Comparisons of the bELISA method with the commercial ELISA kit.

The bELISA method established in this study and the commercial CSFV detection kit (IDEXX, USA; 99-43220) were used for the simultaneous analysis of 300 clinical samples. Three replicates of each serum sample were assayed, and the coincidence rate between the two methods was statistically analyzed.

### Statistical analysis.

Schematics in Fig. 6 were drawn using Adobe Illustrator software (Adobe Illustrator 2020; Adobe, USA), and Origin software (Origin 2020; Origin Lab, USA) was used for data function analysis and plotting. The differences between groups were considered statistically significant if *P* was <0.05. Kappa values were calculated using Statistical Product and Service Solutions software (SPSS; IBM, USA) to estimate the coincidence between the bELISA method and the commercial ELISA kit.

### Data availability.

All data generated or analyzed in this study are included in the article and the supplemental material.
