# Supplementary material for: A Novel Blocking Enzyme-Linked Immunosorbent Assay Based on a Biotinylated Nanobody for the Rapid and Sensitive Clinical Detection of Classical Swine Fever Virus Antibodies
Source: Microbiol Spectr. 2023 Jan 23;11(1):e02996-22. doi: 10.1128/spectrum.02996-22 (PMC9927282; doi:10.1128/spectrum.02996-22)
Supplement: Supplemental file 1 — Fig. S1, Table S1. Download spectrum.02996-22-s0001.pdf, PDF file, 1.6 MB [file spectrum.02996-22-s0001.pdf]

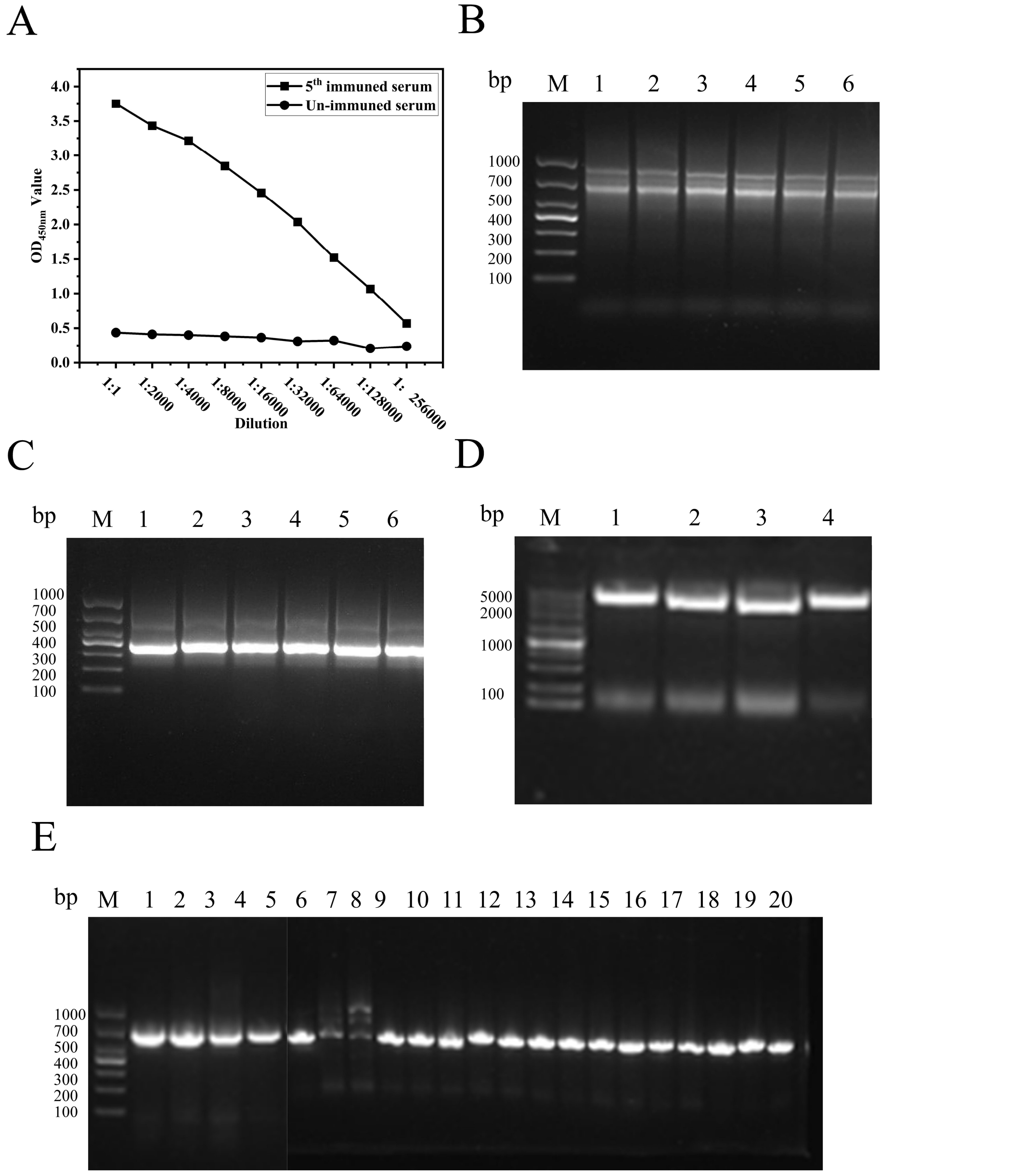

Fig. S1 VHH library construction. (A) Blocking rate for serum after the fifth immunization. (B) PCR amplification products (first round). (C) PCR amplification products (second round). (D) Preparation of the recombinant phage expression vectors. (E) PCR results from the bacterial solution.

Table S1 Specific primer sequences

Pathogen	Label	Sequence (5'-3')
CALL	F1	GTCCTGGCTGCTCTTCTACAAGG
	R1	GGTACGTGCTGTTGAACTGTTCC
VHH	F2	CAGGTGCAGCTGCAGGAGTCTGGGGGAGR
	R2	CTAGTGCGGCCGCTGAGGAGACGGTGACCTGGGT
p5E	F3	AATACGCAAACCGCCTCTCC
